# The Dinoflagellate *Lingulodinium polyedrum* Responds to N Depletion by a Polarized Deposition of Starch and Lipid Bodies

**DOI:** 10.1371/journal.pone.0111067

**Published:** 2014-11-04

**Authors:** Steve Dagenais Bellefeuille, Sonia Dorion, Jean Rivoal, David Morse

**Affiliations:** Institut de Recherche en Biologie Végétale, Département de Sciences Biologiques, Université de Montréal, Montréal, Québec, Canada; University of Connecticut, United States of America

## Abstract

Dinoflagellates are important contributors to the marine phytoplankton and global carbon fixation, but are also infamous for their ability to form the spectacular harmful algal blooms called red tides. While blooms are often associated with high available nitrogen, there are instances where they are observed in oligotrophic environments. In order to maintain their massive population in conditions of nitrogen limitation, dinoflagellates must have evolved efficient adaptive mechanisms. Here we report the physiological responses to nitrogen deprivation in *Lingulodinium polyedrum*. We find that this species reacts to nitrogen stress, as do most plants and microalgae, by stopping cell growth and diminishing levels of internal nitrogen, in particular in the form of protein and chlorophyll. Photosynthesis is maintained at high levels for roughly a week following nitrate depletion, resulting in accumulated photosynthetic products in the form of starch. During the second week, photosynthesis rates decrease due to a reduction in the number of chloroplasts and the accumulation of neutral lipid droplets. Surprisingly, the starch granules and lipid droplets are seen to accumulate at opposite poles of the cell. Lastly, we observe that cells acclimated to nitrogen-depleted conditions resume normal growth after addition of inorganic nitrogen, but are able to maintain high cell densities far longer than cells grown continuously in nitrogen-replete conditions.

## Introduction

Nitrogen (N) is an essential nutrient for all living organisms as it is required for biosynthesis of proteins and nucleic acids. While N is highly abundant in the atmosphere as dinitrogen gas (N_2_), this chemical form is inaccessible to most organisms, which thus depend on diazotrophs, prokaryotes able to transform N_2_ to bioavailable N forms. As a result, demand for N often exceeds its supply, which limits growth in many ecosystems. This is particularly severe in the open oceans where diverse phytoplanktonic species must compete for small amounts of bioavailable N and be able to survive periods of natural N depletion [1].

Dinoflagellates are important members of the phytoplankton that have developed a variety of strategies to cope with N stress [2]. These strategies have a particular importance for those dinoflagellate species able to form harmful algal blooms (HAB), or “red tides”. Some HAB can produce toxins, which can have a strong negative impact on other parts of the ecosystem and on human health. Strategies allowing blooms to deal with N stress might appear counterintuitive, as natural and anthropogenic N addition rather than N deprivation is a factor often associated with HAB [3], [4]. However, in some cases dense blooms can form and persist in oligotrophic environments where the measured inorganic N supply would seem to be insufficient to support their biomass, as was observed for *Karenia brevis* in the West Florida Shelf [5]. In this particular example it was proposed that organic N coming from decaying fish killed by the HAB explained the persistence of the dinoflagellate populations [5], [6]. However, another possibility is that acclimation mechanisms triggered by N deprivation during a bloom could allow the dinoflagellates to maintain bloom-density population levels until new N becomes available. Unfortunately, testing this hypothesis in the environment would be difficult, because of the complex interplay between biotic and abiotic factors involved in bloom dynamics [4].

*Lingulodinium polyedrum* is a marine dinoflagellate usually studied as a model system in circadian biology, but is known to form HAB in various regions of the world, particularly along the coast of Southern California [7], [8], [9]. Laboratory cultures of this species can be readily transferred to media of different composition after filtration, which makes it a good candidate to test the effects of controlled nutrient conditions on its biochemistry and physiology. Interestingly, *L. polyedrum* grown in N-deprived artificial sea water was reported to survive up to 4 times longer than when grown in the normal nitrate (NO_3_^-^) enriched f/2-medium [10]. These findings implied that this species was able to acclimate to N limitation, but the underlying mechanisms were not evaluated. In this study, we have addressed this issue by monitoring the physiological responses of *L. polyedrum* to N stress. In particular, we note that cells acclimated to N limitation rose to high cell densities when new inorganic N was supplied and these for durations normally observed in *L. polyedrum* blooming populations in the environment. This is in sharp contrast to the behavior of non acclimated cells, which abruptly die after reaching their maximum cell density. We also note an accumulation of reduced carbon (C) in the form of starch granules and lipid bodies in N-limited cells. Curiously, these two forms accumulated in different regions of the cell.

## Materials and Methods

### Cell culture

Unialgal but not axenic *Lingulodinium polyedrum* (CCMP 1936, previously *Gonyaulax polyedra*) was obtained from the Provasoli-Guillard National Center for Marine Algae and Microbiota (East Boothbay, ME, USA). Cell cultures were either grown in normal f/2 medium prepared using Instant Ocean (termed day 0) or in f/2 lacking added N (f/2-N) for one or two weeks (termed day 7 or day 14). Day 0 cells acted as control during all experiments. To transfer cells to f/2-N (N-depleted medium), cultures grown in f/2 were filtered on Whatman 541 paper and washed with 200 ml of f/2-N medium before resuspension in f/2-N medium. Normal f/2 medium is made from Instant Ocean supplemented with 0.88 mM NO_3_, and Instant Ocean alone contains 1 µM NO_3_, 10 µM NH_3_ and 3 µM dissolved organic N [11]. All cultures were grown under a 12 h light (40 µmol photons m^−2^ s^−1^ cool white fluorescent light) and 12 h darkness at a temperature of 18±1°C. This light/dark regime is termed LD, with LD 0 corresponding to lights on and LD 12 to lights off. Cells used for elemental analysis, the quantification of total proteins, amino acids, starch and neutral lipids were harvested by filtration and stored at −80°C until use.

### Cell density measurements

Cells in multiple 10 µl aliquots of f/2 and f/2-N cultures were placed on microscope slides and were counted every 3 or 4 days using a Leica Wild M3B stereo microscope. After 17 days, a time when the cultures had typically reached their maximum cell density, 880 µM of NaNO_3_^-^ was added to each culture. Counts were continued until no cells remained swimming in the medium.

### Elemental analysis

Measurements of total C and N contents were performed using a Carlo Erba NC2500 Elemental Analyzer (at Geotop-UQAM, Montreal, PQ, Canada). The analytical step was preceded by a preparative step, where cells were harvested, lyophilized, weighed and inserted into tin capsules. Results are reported as percent of total dry weight.

### Protein and amino acid quantification

For total protein quantification, cultures grown in f/2 were split into f/2 and f/2-N cultures, and samples were taken before, as well as 3, 7, 10 and 14 days after the split. Protein from 30 mg wet weight of cells was extracted following a slightly modified Trizol (Life technologies) protocol as described previously [12]. Protein pellets were resuspended in 7 M urea, 2 M thiourea, 4% CHAPS and 20 mM dithiotreitol, and incubated overnight to completely solubilize the pellets. Protein concentrations were measured using Bio-Rad (Bradford) Protein Assay following the manufacturer's protocol.

Amino acid quantification was performed by HPLC using a protocol previously optimized for plant tissues [13]. Samples were lysed mechanically in 80% (v/v) ethanol for 2 min at 4°C in a bead beater (BioSpec products). They were then extracted at 70°C in 80% (v/v) ethanol and fractionated into neutral, anionic and cationic fractions by preparative ion exchange purification [14]. The cationic fractions were aliquoted and evaporated to dryness before derivatization with the AccQ Fluor reagent kit (Waters). The amino acids in the aliquots were analyzed on a Waters HPLC system controlled by the Empower Pro software and equipped with a 600 controller, a 717 Plus refrigerated automatic sample injector, a 2996 Diode Array Detector and an AccQ.Tag Amino Acid Analysis Column (Waters). HPLC parameters were set according to manufacturer's recommendations. Quantification was done using standard curves from commercial amino acids.

### Photosynthetic measurements

Chlorophyll *a* content was measured after extraction of 50 mg wet weight of cells in 100% acetone. The pigment-containing supernatants were collected after centrifugation and cell residues were re-extracted with small volumes of acetone until colorless. Supernatants were combined and chlorophyll optical densities were measured using a Cary 100 Spectrophotometer (Varian). Concentrations were calculated using the equations presented by [15].

Photosynthetic C fixation rates in normal and N-depleted cultures were calculated from incorporation of ^14^C to an acid-soluble form. For each assay, 1.48 kBq ( = 0.04 µCi) of radiolabelled bicarbonate (NaH^14^CO_3(aq)_, ICN, 310 MBq mmol^−1^) was added to 5 mL of cell culture with dissolved inorganic carbon ≈2 mM [16] and the culture incubated for 90 min under normal lighting (40 µmol photons m^−2^ s^−1^ cool white fluorescent light). The ^14^C labeling was quenched by adding HCl to a final concentration of 1 N and gaseous radioactivity was allowed to escape from unsealed samples vials during an overnight incubation in a fume hood. Samples were transferred into scintillation vials with 2 mL Scintiverse (Fisher Scientific) then counted in a TriCarb 2800TR scintillation counter (Perkin Elmer). Values were reported as the number of disintegrations per minute (DPM) from light-induced C fixation after subtraction of the number of DPM for identical subcultures incubated in the dark. Samples were normalized to cell number.

### Starch quantification

For starch analysis, cells from all cultures were harvested at both LD 0 and at LD 12 and were lysed mechanically in 80% (v/v) ethanol for 2 min at 4°C in a bead beater (BioSpec products). Starch was assayed enzymatically as described [17]. Briefly, the procedure included the removal of soluble sugars with 80% (v/v) ethanol washes at 70°C, the solubilization of starch in 1 M KOH and its conversion into glucose by amyloglucosidase. The glucose concentration was determined using hexokinase (HK) to phosphorylate the glucose and glucose-6-phosphate dehydrogenase (G6PDH) to convert the glucose-6-phosphate to 6-phosphogluconolactone and simultaneously reduce NAD^+^ to NADH. This reduction was monitored spectrophotometrically at 340 nm with the amount of NADH produced proportional to the amount of glucose in the samples.

### Nile red quantification of neutral lipid

Neutral lipids were quantified with Nile red using a fluorometric assay previously optimized for various microalgae [18]. Two mg wet weight cell pellets were resuspended in 25% dimethyl sulfoxide (DMSO) in 1.5 mL tubes with glass beads (0.5 mm, BioSpec products) and were lysed mechanically for 2 min at 4°C in a bead beater (BioSpec products). Small aliquots of these cell solutions were added to a black 96-microplate and the volume was adjusted to 297 µL with 25% DMSO. Fluorescence was recorded using a SpectraMax M5 Microplate Reader (Molecular Devices) under high scan control and high PMT detector voltage mode, using emission and excitation wavelengths of 530 nm and 575 nm, respectively. These wavelengths were selected based on the emission/excitation spectra of the triolein standard used for quantification. Three µL of a 50 µg mL^−1^ Nile red solution was added to each well to a final concentration of 0.75 µg mL^−1^ and the plate was incubated at 37°C for 10 min. Fluorescence values after Nile red-staining were recorded and, after subtraction of the pre-stain fluorescence values, were used for quantification using a triolein standard curve.

### Microscopy

Starch was observed in cells from the a culture taken at both LD 0 or LD 12, after fixation in 70% (w/v) ethanol, and staining using a solution containing 0.5% (w/v) I_2_ and 1% (w/v) KI. Stained cells were visualized with a bright-field Axio Imager M1 microscope (Zeiss).

The contrast of all images was adjusted with Adobe Photoshop 8.0 using the same tonal curve to provide better contrast between the black-stained starch and the dark brown cell background.

Chloroplasts and lipid bodies of day phase living cells were visualized with a LSM 5 Duo confocal microscope (Zeiss). Chlorophyll autofluorescence was observed using a UV-diode (405 nm) excitation and a long pass filter (>575 nm) emission. For lipid bodies, 1 mL aliquots were incubated at 18°C in the dark for 20 minutes with Nile red at a final concentration of 10 µg mL^−1^. Stained lipid bodies were observed using an argon laser (excitation at 514 nm) and emissions at 575 and 615 nm. Differential interference contrast (DIC) images were taken at the same time as the fluorescent images. Cells with the ventral side up were selected for imaging so that the optical slices would have a similar appearance with respect to the location of the C-shaped nucleus and the pyrenoids. All microscope parameters used were the same between samples. Nile red images were modified for contrast only using Adobe Photoshop 8.0.

For transmission electron microscopy, cells were harvested at LD 0, fixed with 2% glutaraldehyde in 0.4 M NaCl, 0.05 M PBS (pH 7.2) for 2 h and then washed 3 times in 0.4 M NaCl, 0.05 M PBS (pH 7.2). Fixed cells were dehydrated following standard procedures and embedded in Spurr's resin as recommended by the manufacturer. Thin sections were contrasted with a saturated solution of uranyl acetate in 50% (w/v) ethanol and immediately observed with a JEOL JEM-1010 electron microscope operating at a voltage of 80 keV.

### Statistical analyses

All experiments were run in triplicate (n = 3) and results were presented as means ± SE. Analysis of variance or Student's t-tests were used for all data. Statistical tests were performed using the JMP software (SAS).

## Results

**Figure 1:**
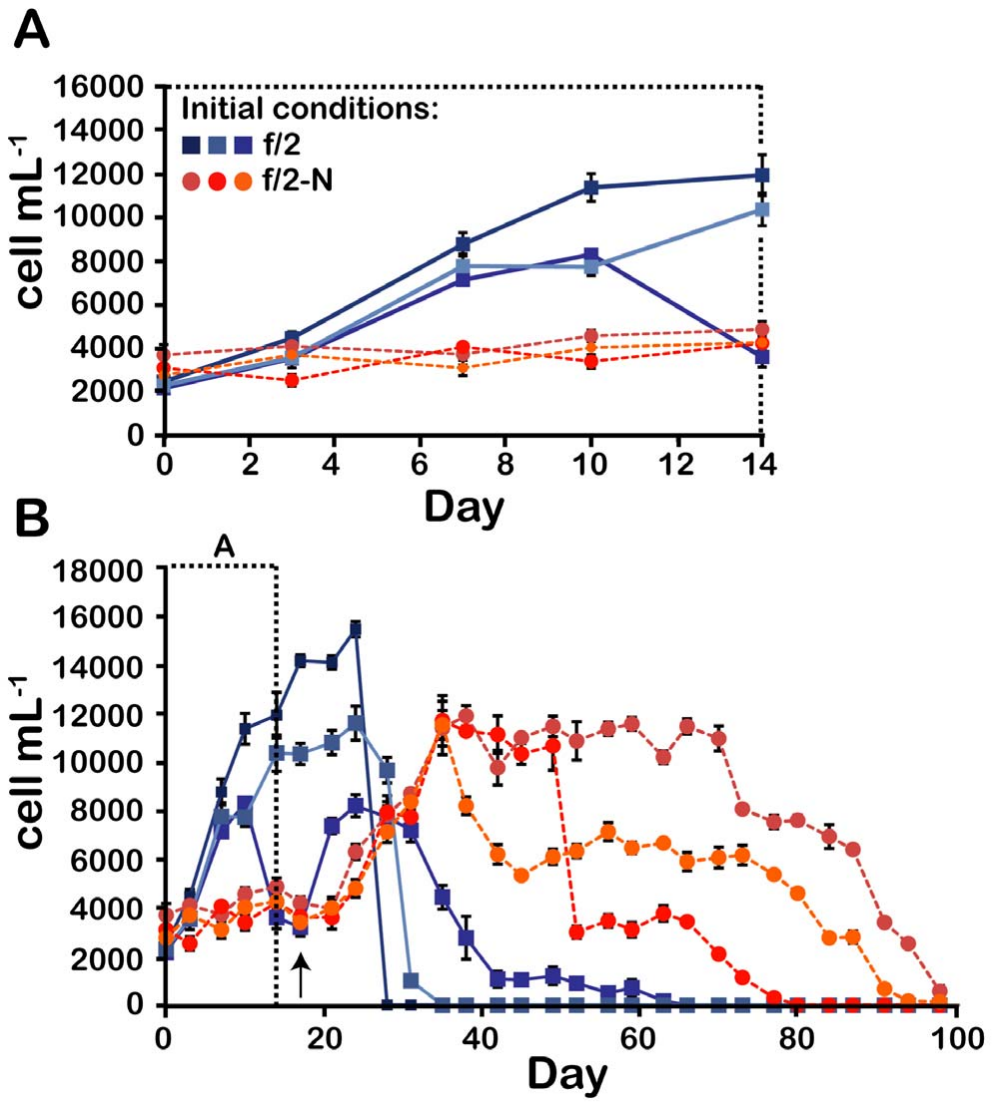
Growth of N-replete and N stressed cultures. The cell density in 6 independent cultures, 3 supplemented with 880 µM NaNO_3_^-^ (f/2) and 3 without added N (f/2-N), was assessed until no swimming cells remained in the culture flasks. The first 14 days are shown on an expanded time scale (A) or over the 100 days of the experiment. The time at which 880 µM NaNO_3_^-^ was added to all cultures is shown by a black arrow. Results are mean ± SE of 3 technical replicates made on cell counts.

To examine the acclimation mechanisms of *Lingulodinium* to N stress, we first determined the physiological responses after transferring N-replete cultures (in standard f/2 medium) to a medium lacking added nitrate (f/2-N). Cultures grown in f/2 medium generally show robust growth for 10–14 days after which the cell densities remain high for several days then drop precipitously (Fig. 1). In some cases, where average growth rates decrease at earlier times, the cultures can survive for slightly longer (Fig. 1B). In contrast, cell growth in f/2-N medium stalled immediately after transfer (Fig. 1A). This inhibition of cell growth is a direct consequence of N depletion, as the addition of nitrate to f/2-N cultures on the 17^th^ day restarted cell growth (Fig. 1B). Interestingly, some cultures acclimated to N depletion demonstrated an ability to survive at high cell densities far longer than cells grown in the N-replete f/2 medium (Fig. 1B). A similar robustness in culture lifetime was also observed for cases where cell densities declined to intermediate values. The cultures acclimated to low N thus appear able to avoid the precipitous collapse of cell densities typically observed for cultures grown in N replete conditions.

**Figure 2:**
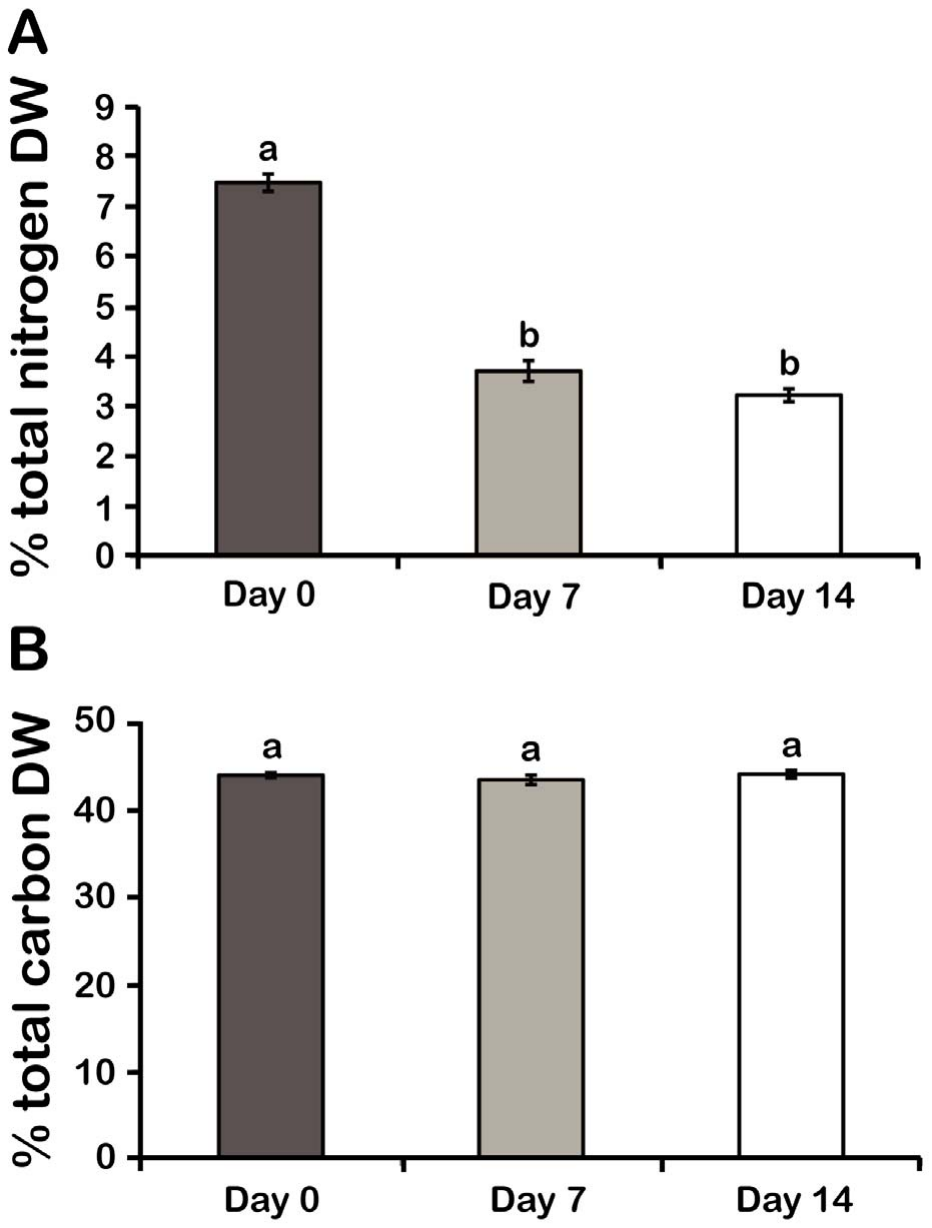
Elemental analysis shows a decreased N content in N stressed cells without a change in their C content. (A) Dry weight percent of total N and B) total C. Results are mean ± SE (n = 3). Statistically different results (p<0.05) are marked with a different letter (Analysis of variance). DW; Dry weight.

Elemental analysis revealed that proportion of cell dry weight as N for cultures grown in f/2-N decreased to almost half their original value after 7 or 14 days (Fig. 2A), while the proportion of cell dry weight as C remained unchanged (Fig. 2B). As these values represent the fractional dry weight allocated to a particular element rather than the absolute amount of that element, the smaller percentage of N observed in f/2-N-grown cells implies that the absolute amounts of other elements are increasing while absolute cellular N levels remain constant.

**Figure 3:**
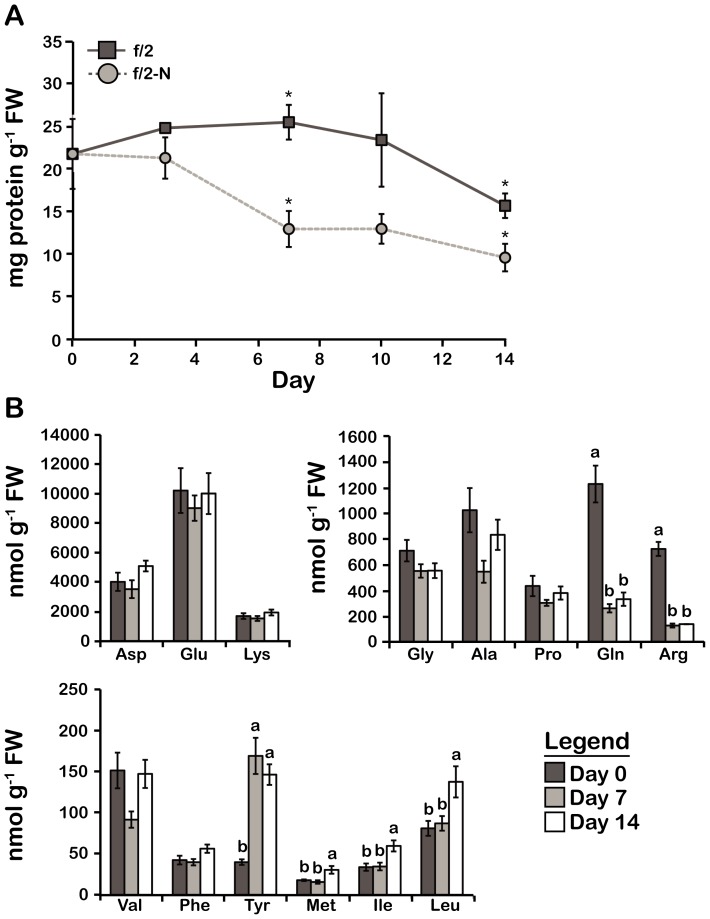
Changes in the total protein content and free amino acid profile in N stressed cells are consistent with a decrease in N assimilation. A) Total protein content. B) Free amino acids, classified into three groups based on their relative abundance. Results are mean ± SE (n = 3). Statistically different results (p<0.05) are marked using either a different letter (Analysis of variance) or an asterisk (Student's t-test). FW; Fresh weight.

The bulk of cellular N is normally sequestered in proteins, and so a reduction in the proportion of N should be accompanied by a reduction in the proportion of protein. To test this, protein levels were measured as a function of time for cultures growing in f/2 and f/2-N media. After 7 days in f/2-N, the protein concentration declined to half the value of the f/2-cultures and remained roughly constant for the following days (Fig. 3A), consistent with the decrease observed for elemental N. The decrease in protein observed for cells grown in normal f/2 medium after 14 days may involve a decrease in protein synthesis rates such as observed in stationary phase cells of *Escherichia coli* [19]. A consequence of this, should protein degradation rates remain unchanged, would be a decrease in protein levels. However, we do not know what signals or environmental factors cause the transition to stationary phase in *Lingulodinium*.

To test if the changes in protein levels observed for f/2-N cultures were also reflected in the free amino acid (FAA) pools, we next characterized the FAA profile of N-depleted cultures using a previously described HPLC-based protocol [13]. Of the 14 FAAs that were quantified by this method, only Gln and Arg levels were found to decrease after N deprivation consistent with the requirement of NH_3_^+^ for their biosynthesis. Most of the FAAs measured, in particular the most highly abundant ones, did not vary significantly during N stress. However, levels of several of the less abundant FAAs, including Tyr, Met, Ile and Leu, were observed to increase after 7 or 14 days in f/2-N. When the FAA data are considered as a whole, the total N in FAA shows no significant change between f/2 and f/2-N cultures (Fig. S1A, S1B). However, the calculated Gln/Glu ratio, considered a useful indicator of N assimilation rates [20] was ∼4 times lower in N-deplete than in N-replete cells (Fig. S1C). Thus, despite a constant total FAA pool, N-depleted cultures show the effect of a severe decrease in N assimilation.

**Figure 4:**
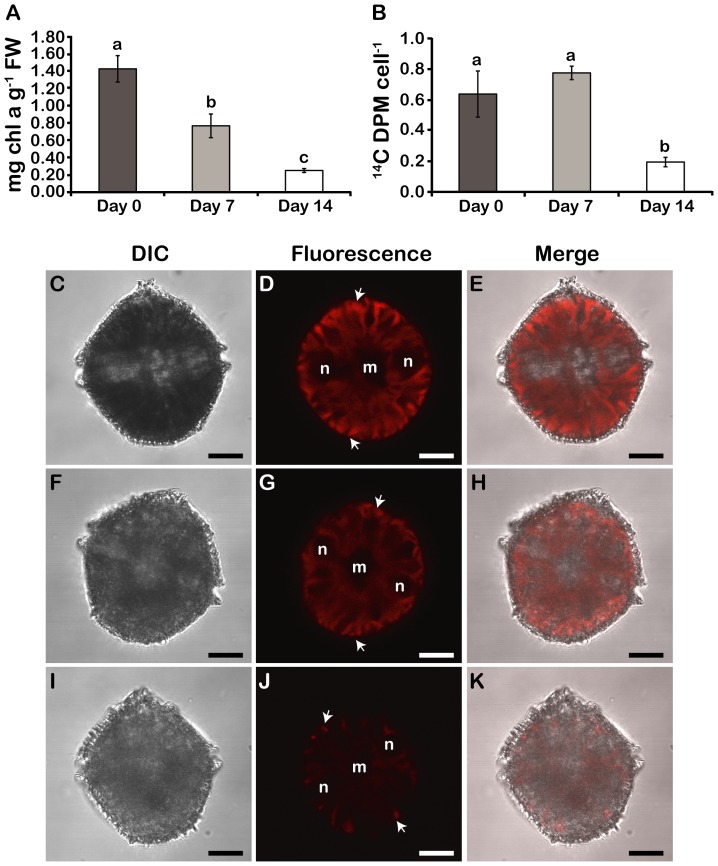
Photosynthesis decreases in N stressed cells. A) Chlorophyll *a* levels. FW; Fresh weight. B) Photosynthetic rates of ^14^C fixation. Results are mean ± SE (n = 3). Statistically different results (p<0.05) are marked with a different letter (Analysis of variance). (C–K) DIC, chlorophyll autofluorescence and merged images of day phase cells at day 0 (C–E), day 7 (F–H) and day 14 (I–K). Chlorophyll intensity is highest in the periphery of day phase cells cells (white arrows) due to a separation of Rubisco and the light harvesting peridinin-chlorophyll *a*-protein within individual chloroplasts (Nassoury et al., 2001). All cells were pictured from a ventral view. In this orientation, two ends of the C-shaped nucleus (n) surround the centrally located ER and Golgi membranes (m). Scale bars are 10 µm.

When compared to the f/2-cultures, chlorophyll *a* concentrations gradually diminished by two- and six-fold by 7 and 14 days of incubation in f/2-N, respectively (Fig. 4A). However, a concomitant decrease in the rate of photosynthesis, as measured by ^14^C-incorporation in acid-soluble compounds, was only observed after 14 days in N deprivation (Fig. 4B), with CO_2_ fixation rates actually showing an increase after 7 days N deprivation. One possible explanation for this is that the initial decrease in chlorophyll aids C fixation by decreasing O_2_ evolution rates [21], but by 14 days the ability of the cells to produce photosynthetic reductant is no longer sufficient to maintain high CO_2_ fixation rates. Interestingly, observation of chlorophyll autofluorescence in living cells by confocal microscopy (Fig. 4C–K) supports the steady decrease in chlorophyll levels during N- stress from 7 (Fig. 4G) to 14 days (Fig. 4J). Cells observed after 14 days also show a decreased number of chloroplasts (Fig.4J).

**Figure 5:**
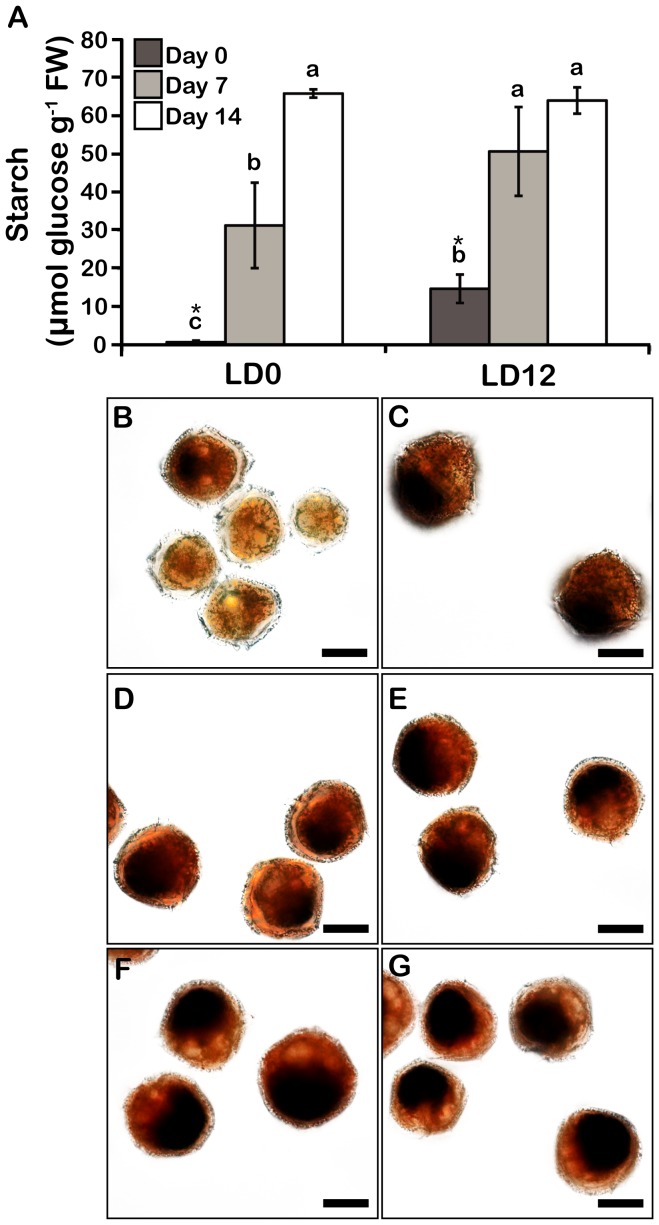
Starch accumulates in N stressed cells. A) Starch levels in cells harvested at LD 0 and LD 12. Results are mean ± SE (n = 3). Statistically different results (p<0.05) are marked using either a different letter (Analysis of variance) or an asterisk (Student's t-test). FW; Fresh weight. Bright-field microscopy of iodine-stained starch granules at day 0 (B–C), day 7 (D–E) and day 14-cells (F–G) at either LD 0 (B, D, F) or LD 12 (C, E, G). Starch is localized at the posterior part of the cells.

The ability of the cells to maintain high levels of C fixation for at least one week in N-deplete conditions suggested that reduced C might accumulate in these cells. To test this, we assessed the levels of starch and neutral lipids, two C pools previously reported to increase during N stress in other dinoflagellates and microalgae [22], [23], [24], [25]. *L. polyedrum* cultures show a clear increase in starch levels after 7 and 14 days of N depletion (Fig. 5A). In the leaves of higher plants, starch is synthesized during the day and degraded at night [26], and the data show that a similar rhythm is found in *L. polyedrum* grown in the presence of N. In these cells, starch levels were substantial at dusk (LD 12) yet almost undetectable at dawn (LD 0). However, this daily variation was abolished in N-deplete cells after 14 days, suggesting the reduced C stores are not being used for metabolism. Curiously, a higher variation in starch levels was repeatedly observed in cells grown for 7 days in f/2-N and measured at LD 0 indicative of a greater heterogeneity in starch metabolism at that stage. Starch accumulation can also be visualized microscopically using iodine to stain the starch granules (Fig. 5B–G). Most of the cells at day 0 did not contain starch at the end of the night (LD 0), but contained substantial amounts at the end of the day (LD 12) (Fig. 5B, C). In addition, higher levels of starch accumulate in N-deplete cultures (Fig. 5D–G). Intriguingly, starch granules always accumulated at the posterior end of the cells. This suggests that starch is not localized within chloroplasts, as these organelles are distributed over the entire cell. A cytosolic localization of starch granules has been reported in other dinoflagellates, in contrast to the plastidic localization seen in land plants and green algae [27], [28], [29].

**Figure 6:**
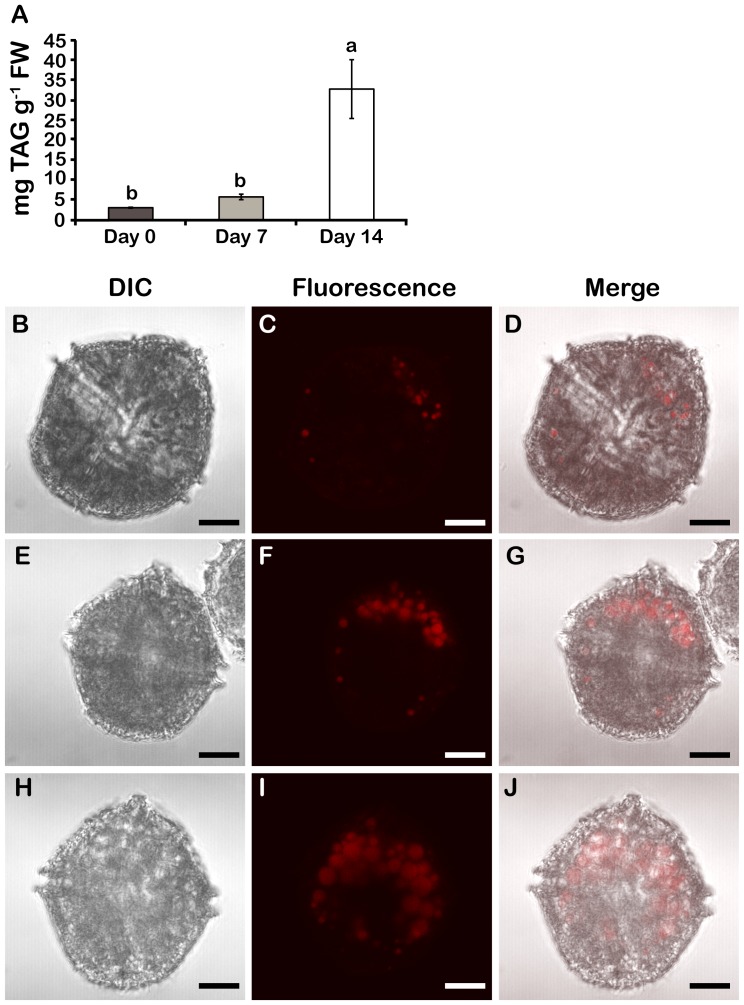
TAGs accumulate in N stressed cells. A) Neutral lipid levels. Results are mean ± SE (n = 3). Statistically different results (p<0.05) are marked with a different letter (Analysis of variance). FW; Fresh weight. DIC, Nile red-stained lipid bodies and merged images of day 0 (B–D), day 7 (E–G) and day 14 cells (H–J). All cells were pictured from a ventral view. Lipid bodies were most predominant in the anterior part of the cells.

Neutral lipids, particularly triacylglycerols (TAGs), also accumulate in N-deplete *Lingulodinium* (Fig. 6). TAG levels in cell extracts, measured using Nile red, were ∼2 times and ∼10 times higher than in cells at day 0 after 7 and 14 days in f/2-N, respectively (Fig. 6A). Again, this form of C storage can be visualized in cells microscopically (Fig. 6B–J). Abundance and size of lipid bodies clearly increased with the duration of N stress (Fig. 6C, F, I). However, in sharp contrast to the starch granules, TAGs accumulated preferentially at the anterior end of the cell (Fig. 6D, G, J).

**Figure 7:**
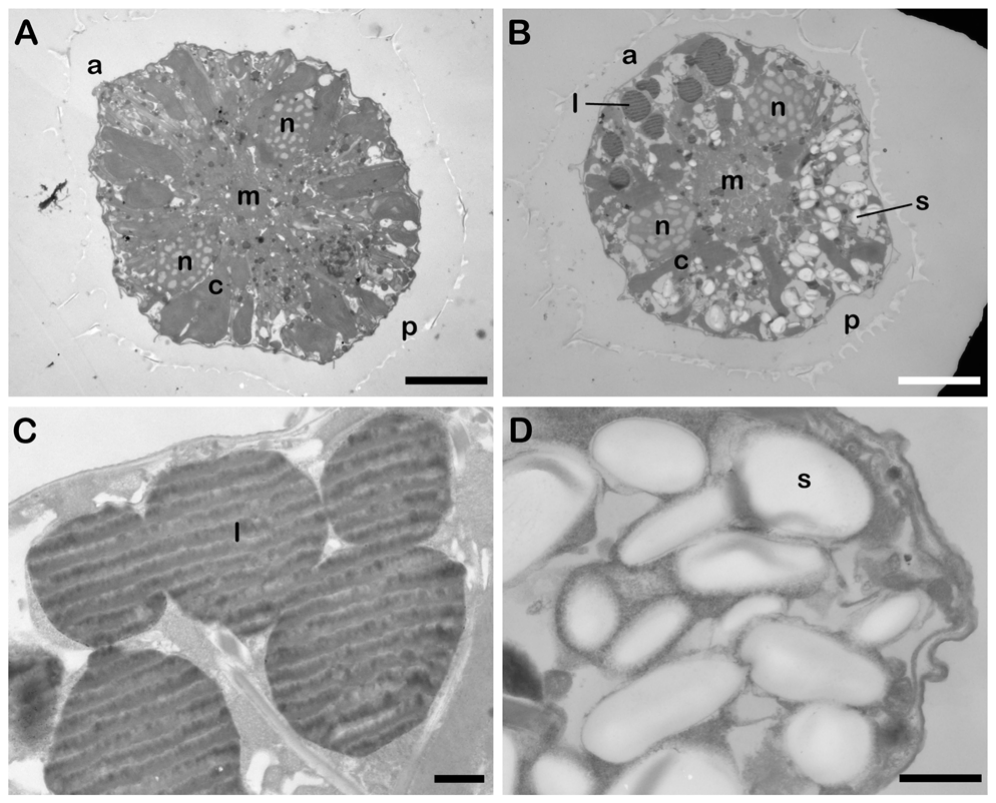
Polarized localization of lipid bodies and starch granules visualized by transmission electron microscopy. Cross-sections of a cell at day 0 (A) and at day 14 in f/2-N medium (B). Both are in a ventral orientation and scale bars are 10 µm. Lipid bodies (l) are located predominantly at the anterior (a) end of the cell, while starch granules (s) are localized at the posterior (p) end. The ends of the C-shaped nucleus (n) surround a central Golgi/ER membrane region (m). Chloroplasts (c) are less abundant in day 14 cells. Higher magnification images of lipid bodies (C) and starch granules (D) in a cell at day 14. Scale bars are 1 µm.

To confirm the asymmetrical distribution of TAGs and starch granules, cells were examined using transmission electron microscopy to visualize both types of C stores in the same cell (Fig 7). Cells at day 0 and day 14 cells were fixed at LD 0, a time at which neither lipid bodies nor starch were observed in the day 0 cell (Fig. 7A). However, the two types of C stores have clearly accumulated at opposite ends of the cell by 14 days in f/2-N medium (Fig. 7B). Lipid bodies are located at the anterior end and appear dark due to the lipophilic nature of the osmium tetroxide stain used to contrast the sections (Fig. 7C) while starch granules appear white and are located at the posterior end of the cell (Fig. 7D). The white striations observed in the lipid droplets are likely due to a sectioning artifact, as their orientation is the same for all cells in a section independent of how the individual cells are orientated in the section. Lastly, we also note that chloroplasts appear smaller and are less abundant in the day 14 cell when compared to the day 0 cell, in agreement with the confocal images (Fig. 4D, G, J).

## Discussion

In this study, we show that *Lingulodinium polyedrum* cultures with cell densities similar to those observed in blooming populations in the field (∼10^7^ cells L^−1^), are able to survive and maintain these elevated cell densities for more than 2 weeks if previously acclimated to N-deplete conditions (Fig. 1) [30]. The cells cease cell division upon transfer to the N-depleted medium, a response to N limitation commonly observed in other dinoflagellates, plants and algae [31], [32], [33], [34], [35], [36]. The arrest of growth in f/2-N was immediate, not gradual, and suggests that *L. polyedrum* grown in a NO_3_^—^replete medium does not store N in a form that could have sustained growth and division under N stress. This differs from the N storage observed in *Symbiodinium* spp. where uric acid crystals accumulate following increases in environmental N [37]. The crystalline inclusions rapidly dissolve, but it has been suggested that the resulting N is remobilized for long-term storage in other forms within the cytosol. Either *L. polyedrum* does not share this long-term storage ability with *Symbiodinium* or its storage capacity is insufficient to support cell division under N limitation. Another possibility is that sensors at the *Lingulodinium* plasma membrane, similar to the CHL1 nitrate transporter in *Arabidopsis*, detected an absence of extracellular NO_3_^-^ and signaled a stress response, which included an early arrest of the cell cycle [38].

C and N metabolism are normally coupled because synthesis of amino acids and nucleotides is dependent upon C skeletons and energy provided by photosynthesis [35]. During N depletion, *L. polyedrum* was restricted in its ability to assimilate N, while its photosynthetic machinery was, for at least a week, still fully functional. This is indicated by the ^14^C fixation rates, which only showed a significant decrease after 14 days of N deprivation (Fig. 4B). We propose that during their first week of N-limitation, the cells divert their excess C toward the synthesis of starch and neutral lipids (Fig. 5; 6). Because neither of these storage compounds contain N, this would result in the decrease in the proportion of N in these N-limited cells that we have observed (Fig. 2A). Furthermore, as the proportion of C in sugar and TAG is roughly 40% C and 75% C, respectively, it seems reasonable that a mixture of both could provide a means of increasing the mass of the cell through new C fixation while at the same time maintaining a constant proportion of C. However, the situation after 14 days in N-limited condition differs. Importantly, the rates of C fixation have decreased markedly (Fig. 4B), in agreement with the small increase in starch observed (Fig. 5A). Despite this, we observe a pronounced increase in TAG (Fig. 6A). We suggest that the accumulation of TAG may result from a recycling of the membranes associated with the chloroplast, whose size and numbers have both decreased in 14 days N-limited-cells (Fig 4J). Therefore, the acclimation of cells to N-limited conditions appears to show a biphasic metabolic response, with an initial maintenance of photosynthesis producing primarily starch as accumulated C, and a subsequent recycling of chloroplast membranes resulting in an increased TAG accumulation.

The proportional reduction in elemental N content is mirrored by the decrease in cellular protein (Fig. 3A) since protein is the most important pool of cellular N in the cell. In contrast, the total FAA levels remained unchanged (Fig. S1A). Thus, since the mass of the cells increases as a result of ongoing photosynthesis, this implies that the absolute amount of FAA in the culture increases during N limitation in order to maintain the same levels relative to the cell mass. One possibility is that the extra FAA result from protein degradation. However, it seems unlikely that degradation of proteins would change the relative abundance of the FAA pools, suggesting that some of the variations may reflect other causes. For example, the lack of available N for ammonium synthesis seems the likely explaination for the decrease observed in Gln and Arg levels (Fig. 3B). In addition, the levels of some FAA, in particular those derived from pyruvate (such as Leu and Ile) or phospho*enol*pyruvate (such as Tyr or Phe) might be augmented using excess C skeletons originating from glycolysis (Fig. 3B). An increase in the levels of these glycolytic intermediates might in turn result from a decreased activity of the tricarboxylic acid cycle under N limitation where energy requirements are likely to be lower than in actively dividing cells. However, additional studies investigating levels of both glycolytic and TCA cycle intermediates would be required to test these possibilities.

N limitation in *L. polyedrum* causes a gradual decrease in chlorophyll *a*, in agreement to what is observed in other photosynthetic eukaryotes [35], [39]. It is unlikely that the dinoflagellate had difficulties in synthesizing new chlorophyll molecules in N-deplete conditions, since glutamate, the precursor for the porphyrin moiety in chlorophyll, remained stable under N stress (Fig. 3B). However, many different N-limited algal cells are shown to be more susceptible to photoinhibition in normal light conditions than N-replete cells [40]. In fact, levels of the D1 protein are markedly lower when the cells are N-limited than when N-replete. D1 is a component of the photosynthetic reaction center in PSII and is proposed to be the site of photodamage. It has been suggested that photoinhibition occurred at lower irradiance in N-limited cells, because the lower rates of protein synthesis are unable to keep pace with degradation of damaged D1 [40]. In our study, it is likely that *L. polyedrum* diminishes its chlorophyll content during N depletion to prevent photooxidative damage, because a similar susceptibility to photoinhibition has been previously reported for this species [41].

The reduction in chlorophyll levels, and the resulting protection against oxidative stress, thus appears distinct from the reduction in the size and number of the chloroplasts observed by 14 days in N limiting conditions (Fig. 4J). The decrease in chloroplast number and size during N stress is suggestive of autophagy, the process by which an organism degrades its own cellular components in order to recycle nutrients or eliminate damaged material. Interestingly, autophagy is important for the survival of *Arabidopsis* and *Chlamydomonas* in conditions of N limitation [42], [43]. In the latter organism, N stress causes the polar lipids of plastid membranes to be recycled for the production of TAGs [44], [45]. We propose that initially, neutral lipids could be produced *de novo*, fueled by C fixation, while at latter stages during N-limitation, TAG accumulation reflects autophagy of chloroplast membranes.

Interestingly, a recycling of plastid membranes might also explain the polarized distribution of lipid bodies in *L. polyedrum*. This species typically contains a single PAS (Periodic acid-Schiff) body, thought to be a dinoflagellate lysosome, in the posterior end of the cell where the lipid droplets are rare [46]. Thus, if the PAS body was involved in neutral lipid degradation, this might favor an anterior location for the accumulation of lipid droplets. TEM observations show small lipid bodies surrounding the cell center in addition to the larger droplets that accumulate predominantly at the anterior end (Fig. 7B). It is possible that the smaller droplets are associated with lipid bodies that have recently budded off from the ER membranes, centrally localized in this dinoflagellate [47]. This would agree with the ER membranes being the site for the last reactions of TAG biosynthesis. The restricted cellular location of the large lipid droplets could result from the fusion of smaller lipid bodies that would, because of the PAS body, strictly accumulate at the anterior end of the cell. Another intriguing possibility could be that enzymes for TAG biosynthesis are polarized within the cell. Independent of the mechanism for localizing the lipid body, however, their location may be functionally important, as lipids were shown to contribute to cell buoyancy in various planktonic species [48], [49]. For example, an accumulation of lipid droplets at the anterior pole at the beginning of the day could help *L. polyedrum* migration to the water surface.

Starch accumulation is a common response to N limitation, both in plants and green algae, as well as in *L. polyedrum*. However, it is unlikely that these different organisms share a common regulatory pathway, because they synthesize starch in different cellular compartments. In the plastids of plants and green algae, glucose-1-phosphate is activated to ADP-glucose using an ADP-glucose pyrophosphorylase before being transferred by starch synthase to a terminal glucose residue for elongation of a glucan chain [50]. The ADP-glucose pyrophosphorylase is inhibited by inorganic phosphate and activated by the Calvin cycle intermediate, 3-phosphoglycerate [50]. Cytosolic P_i_ is produced by sucrose synthesis and is exchanged with chloroplastic triose-phosphate by the triose phosphate-phosphate translocator [50]. In contrast, if starch is synthesized in the cytosol of dinoflagellates, then no exchange of metabolites across the plastid membranes is required. Furthermore, multiple soluble starch synthases and one granule-bound starch synthase of the heterotrophic dinoflagellate *Crypthecodinium cohnii,* display a marked substrate preference for UDP-glucose rather than ADP-glucose [29]. A similar preference is observed in all organisms storing starch in the cytosol, such as the rhodophytes, glaucophytes and cryptophytes [51], [52], [53]. Thus, starch synthesis in dinoflagellates might proceed through a cytosolic UDP-glucose pyrophosphorylase, but characterization of this enzyme and its regulators will be required to validate this suggestion.

Localization of the enzymes involved in *L. polyedrum* starch biosynthesis is of particular interest, considering the observation that starch granule accumulation is polarized in these cells (Fig 5B-G; Fig. 7B). The asymmetrical distribution is not simply a result of N limitation, because N-replete cells at LD12 accumulate granules at the same position as in N-limited cells. It also seems unlikely that starch could be transported to the posterior end by motor-proteins traveling on cytoskeletal filaments, because we were unable to detect any membrane surrounding the granules (Fig. 7D), in agreement with a previous TEM study of this species [47]. We suggest that starch synthases and starch branching enzymes might be tethered to membranes at the posterior end of *L. polyedrum* and these localized enzymes might provide a scaffold for polarized starch synthesis. Protein immunolocalization could assess this hypothesis, particularly now that sequences of starch metabolic enzymes are likely to be found in the complete transcriptome of *L. polyedrum* [54].

The adaptation to N-deplete conditions allow *L. polyedrum* cells that are re-exposed to N to survive at high cell densities for up to a month instead of the few days that N-replete cultures normally tolerate (Fig. 1B). Thus, during the period of N depletion, the cells have acquired a capacity to resist N stress that N-replete cells do not have. Interestingly, conditions of N stress are more likely to be encountered by this species in the field than continuous exposure to high N such as is the case for usual laboratory culture conditions. This increased endurance could help blooming populations survive and maintain their density for long periods of N stress in the environment. Further studies will be required to determine which molecular adaptations accompany this physiological adaptation.

## Supporting Information

Figure S1 **Sum of all free amino acids (A), sum of N in amino acids (B), and Gln/Glu ratio (C).** The amino acid data were compiled from Fig. 3B.
(DOCX)
