# Supplementary material for: The Dinoflagellate Lingulodinium polyedrum Responds to N Depletion by a Polarized Deposition of Starch and Lipid Bodies
Source: PLoS One. 2014 Nov 4;9(11):e111067. doi: 10.1371/journal.pone.0111067 (PMC4219697; doi:10.1371/journal.pone.0111067)
Supplement: Figure S1 — Sum of all free amino acids (A), sum of N in amino acids (B), and Gln/Glu ratio (C). The amino acid data were compiled from Fig. 3B. (DOCX) [file pone.0111067.s001.docx]

Supplementary material

**
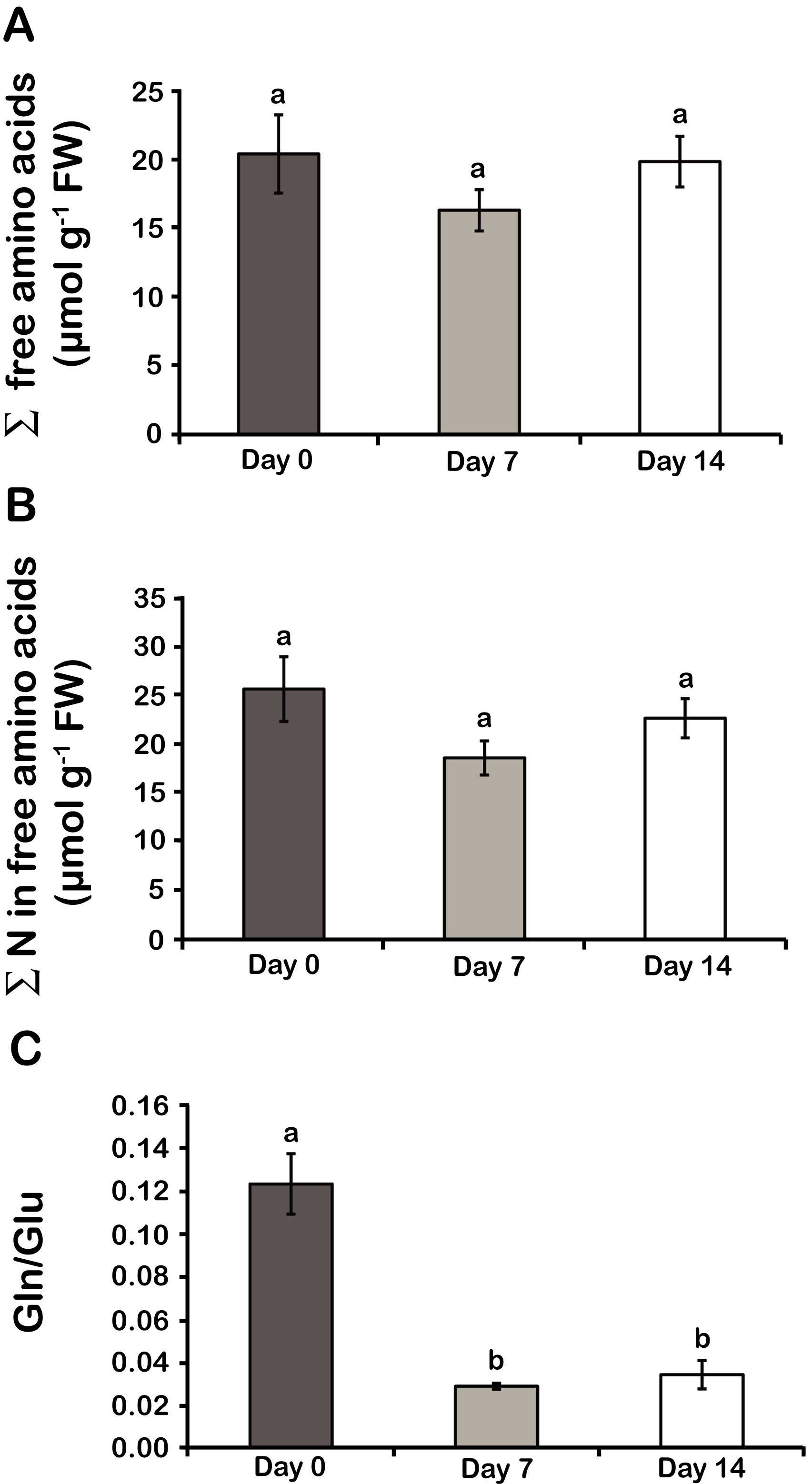
**

**Fig. S1:** A) Sum of all free amino acids. B) Sum of N in amino acids. C) Gln/Glu ratio. The amino acid data were compiled from Fig. 3B.
